# Updating Reaction Mechanistic Domains for Skin Sensitization: 1. Nucleophilic Skin Sensitizers

**DOI:** 10.1021/acs.chemrestox.4c00207

**Published:** 2024-09-11

**Authors:** David W. Roberts, Anne Marie Api, Aynur Aptula, Isabelle Lee, Holger Moustakas

**Affiliations:** † School of Pharmacy and Biomolecular Sciences, Liverpool John Moores University, Byrom Street, Liverpool L3 3AF, England, United Kingdom; ‡ Research Institute for Fragrance Materials, Inc, 1200 MacArthur Boulevard no. 306, Mahwah, New Jersey 07430, United States; § SEAC, Unilever, Colworth Science Park, Sharnbrook, Bedfordshire MK44 1LQ, England, United Kingdom

## Abstract

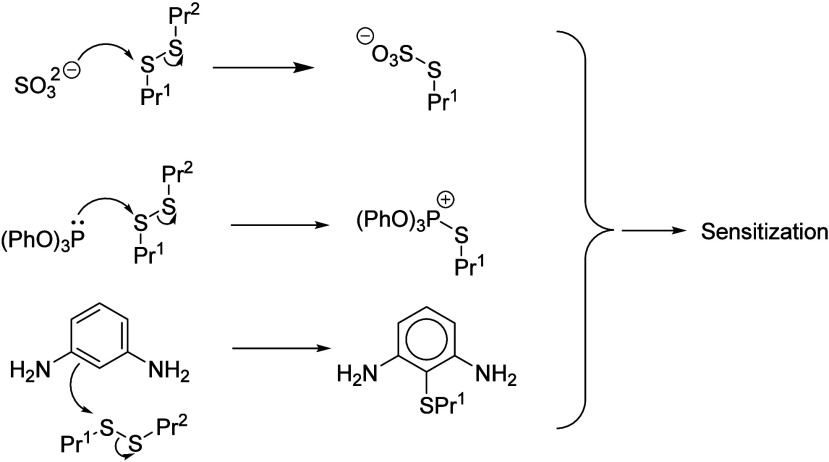

It has long been recognized that skin sensitizers either are electrophilic or can be activated to electrophilic species. Several nonanimal assays for skin sensitization are based on this premise. In the course of a project to update dermal sensitization thresholds (DST), we found a substantial number of sensitizers, with no electrophilic or pro-electrophilic alerts, that could be simply explained in terms of the sensitizer acting as a nucleophile. In some cases, the nucleophilic center is a sulfur or phosphorus atom, while in others, it is an aromatic carbon atom. For carbon-centered nucleophiles, a quantitative mechanistic model based on a combination of Hammett σ^+^ and logP values has been derived. This has been applied to rationalize several groups of known sensitizers with no electrophilic or pro-electrophilic alerts, including anacardic acids and cardols, which are known human sensitizers associated with, inter alia, cashew nut oil, mango, and *Ginkgo biloba*. The possibility of nucleophilic sensitization needs to be considered when evaluating new chemicals for skin sensitization potential and potency by nonanimal assays, particularly those based on the premise that skin sensitization is dependent upon reactions of electrophiles with skin protein-based nucleophiles.

## Introduction

**Figure 1:**
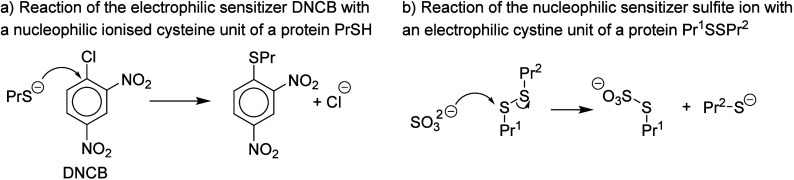
Reactions of (a) an electrophilic sensitizer (DNCB) and (b) a nucleophilic sensitizer (sulfite anion) with cysteine and cystine units of proteins, respectively.

Most known skin sensitizers are either electrophilic or able to be activated to electrophilic species.^1,2^ The molecular initiating event in the AOP (adverse outcome pathway) for skin sensitization is covalent modification of proteins in the skin.^2^ The precise nature and location of these proteins are not known. The covalent modification process is generally regarded as involving an attack of the nucleophilic groups of proteins (e.g., ionized −SH of a cysteine unit, −NH_2_ of a lysine unit) on an electrophilic reaction center of a skin sensitizer, as illustrated in Figure 1 for the well-known sensitizer dinitrochlorobenzene (DNCB). However, there are some cases where the skin sensitizer appears neither electrophilic nor pro-electrophilic.

A mechanism in which the sensitizer acts as the nucleophile and the skin protein is the electrophile has been proposed to rationalize the skin-sensitizing properties of sodium metabisulfite^3^ and phosphite esters.^4^ From this point, we will refer to chemicals proposed to sensitize in this way as nucleophilic sensitizers, and the skin sensitization that they produce will be referred to as nucleophilic sensitization.

In the course of a project updating dermal sensitization thresholds,^1^ we analyzed an expanded data set with skin sensitization data, as determined in the murine local lymph node assay (LLNA). This assay quantifies skin sensitization potency in terms of the EC3 value, the concentration, expressed as percentage by weight, of the test chemical applied to the skin that produces a 3-fold increase in lymphocyte proliferation compared to controls. For quantitative modeling purposes, potency is quantified on a molecular basis as pEC3 (p indicating the operator −log_10_), defined as log_10_(*M*/EC3) where *M* is the molecular weight. In this expanded data set, which consists of 556 sensitizers and 596 nonsensitizers, we found a substantial number of cases where sensitization may be most simply explained in terms of the sensitizer acting as a nucleophile, and some indications of structure–activity patterns for nucleophilic sensitization began to emerge. Here, we report our findings so far.

## Reaction Chemistry Considerations

In order for a nucleophile to sensitize, it must be able to react covalently with an electrophilic group present in skin protein. The most obvious electrophilic groups in proteins are the S–S disulfide linkages of cystine units. These can act as soft electrophiles, as shown in Figure 1. The nucleophile attacks one of the sulfur atoms, and the S–S bond breaks heterolytically, with the thiolate anion of the cysteine unit acting as a leaving group. The effectiveness of the thiolate anion as a leaving group depends inversely on the p*K*_a_ of the corresponding thiol. The SH groups of the cysteine units in proteins have p*K*_a_ values ranging from ∼4 to ∼9 depending on the nature of the neighboring amino acid units in the secondary and tertiary structure (Kortemme and Creighton, 1995 and references therein).^5^

Consequently, some cystine-based disulfide linkages in proteins are likely to be much more reactive than others toward nucleophiles. The reaction chemistry shown in Figure 1 is well established for sodium metabisulfite behaving as the nucleophile, Nu.^6^ It is, at least in part, the basis of the preservative properties of sodium metabisulfite. Harvey et al. observed similar chemistry with trivalent phosphorus esters.^7^

As was previously described, for a nucleophile to act as a skin sensitizer, it needs to be able to react with the S–S linkages of the cystine units in a reaction analogous to that shown in Figure 1 for the sulfite anion. To do this, it needs to be a soft nucleophile, or at least borderline, and sufficiently reactive. Nucleophiles in which the reaction center is divalent sulfur (ionized), trivalent phosphorus, or carbon seem most likely to meet these criteria.

## Sulfur Nucleophiles

**Figure 2:**
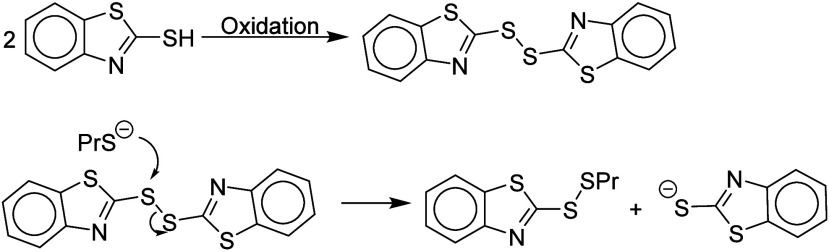
Pro- or pre-electrophilic mechanism proposed for 2-mercaptobenzothiazole (**3**).

Table 1 lists the sulfur compounds we encountered for which sensitization by a nucleophilic mechanism is plausible. For some of these compounds, an alternative pro-electrophile mechanism is also conceivable. Chipinda et al.^8^ reported evidence that compound **3**, 2-mercaptobenzothiazole, can sensitize via its electrophilic disulfide oxidation product (Figure 2), and this seems to be at least equally plausible as the nucleophilic mechanism. This oxidation–electrophilic mechanism can be envisaged on paper for all compounds with an −SH group, as illustrated in Figure 2. For this mechanism to be plausible for a compound, RSH, it requires first that RSH be readily oxidizable to RSSR and second that RS^–^ be a good leaving group, readily displaceable by an ionized cysteine unit. The first requirement is met by most SH compounds and for practical purposes can be taken for granted. To meet the second requirement, leaving group RS^–^ should not be significantly more basic than the incoming nucleophile, the ionized cysteine unit. In other words, RSH should have a p*K*_a_ that is not much greater than that of a cysteine unit. This requirement is met by compound **3**, which has a p*K*_a_ of 6.94.^9^ The SH group in 3-amino-1,2,4-triazole-5-thiol and 2-mercaptobenzimidazole (**10** and **12**) is in a similar environment to that of compound **3**, and the oxidation–electrophilic mechanism seems to be plausible for these compounds also. Compound **2**, sodium diethyldithiocarbamate, also has an acidic SH group. Thus, the oxidation–electrophilic mechanism also seems to be plausible for this compound, which is used as its zinc salt in the manufacturing of rubber and latex products. Chipinda et al.^10^ investigated its haptenation mechanism and suggested that it sensitizes either through metalloprotein chelation or by an electrophilic reaction of one of its oxidation products. It may be noted that **2** as its sodium salt is a stronger sensitizer (EC3 = 1.66, pEC3 = 2.01) than one of the two major electrophilic oxidation products Et_2_N–C(=S)–S–S–C(=S)–NEt_2_ (EC3 = 5.42, pEC3 = 1.73) and weaker (on a molar basis) than the other, Et_2_N–C(=O)–S–S–C(=O)–NEt_2_ (EC3 = 1.70, pEC3 = 2.19). The dimeric oxidation products RO–C(=S)–S–S–C(=S)–OR of xanthate salts, represented here by **8** and **9**, are electrophilic, but oxidants more powerful than molecular oxygen are required to effect the oxidation;^11,12^ therefore the oxidation–electrophilic mechanism is less plausible for compounds **8** and **9** than it is for compounds **2**, **3**, **10**, and **12**. Xanthate salts are good nucleophiles,^13^ so the nucleophilic mechanism for compounds **8** and **9** provides the simplest explanation for their sensitization properties.

**Table 1:**
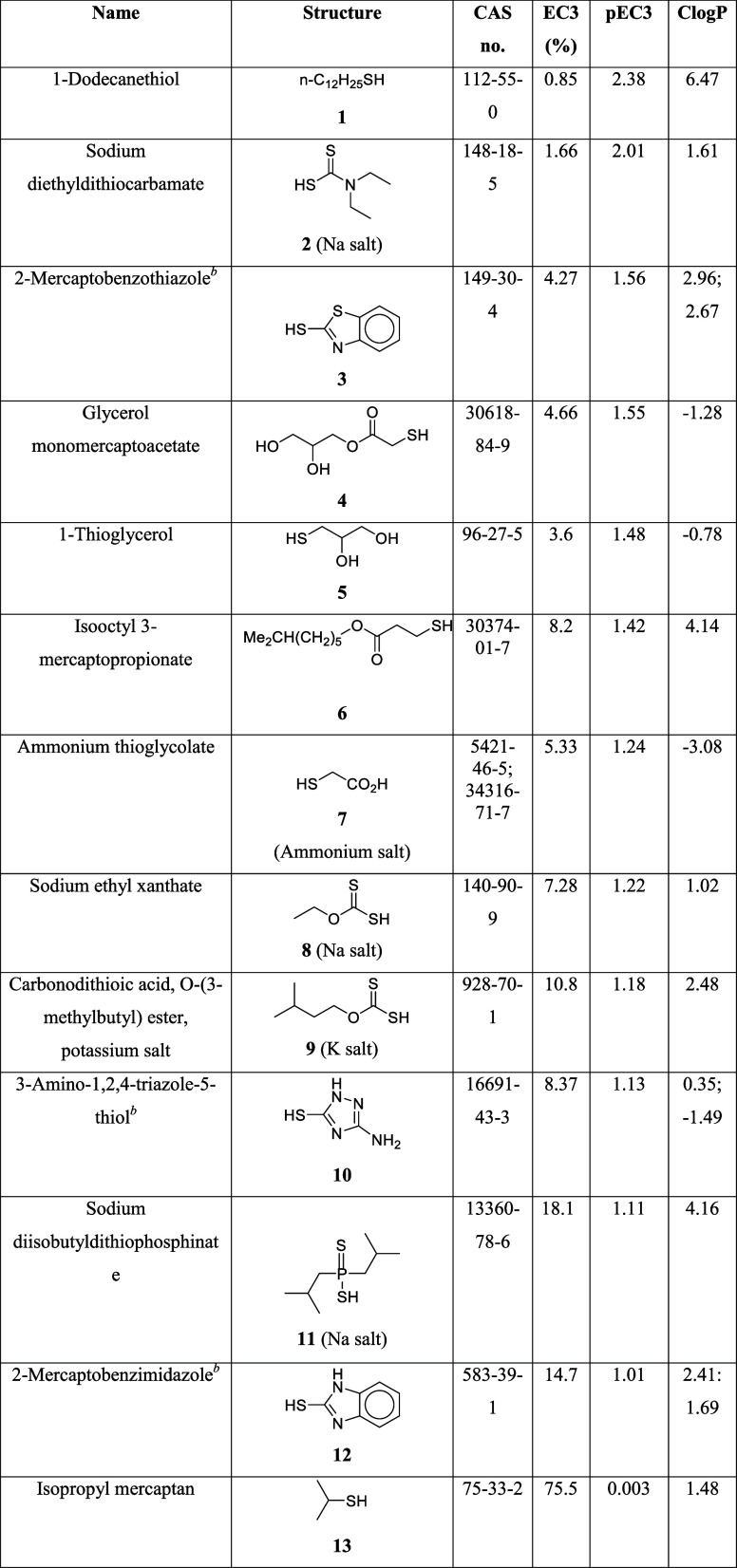
Sulfur Nucleophiles^a^

a The compounds are listed in decreasing order of molecular potency (pEC3 values).

b These compounds can exist in two tautomeric forms: the thiol shown and the cyclic dithiocarbamate (for **3**) or cyclic thiourea (for **10** and **12**). The first ClogP value given is for the thiol shown, and the second ClogP value is for the tautomer with a nominal S=C structure (see Supporting Information SI1). We have not found information as to which tautomer will predominate in cutaneo. We consider it likely that the S=C tautomers will have substantial zwitterionic character which could enable them to act directly as nucleophilic sensitizers (see Supporting Information SI1).

For compounds **1**, **4**–**7**, and **13** the oxidation–electrophilic mechanism seems to be less plausible since the disulfides resulting from oxidation would not be expected to be strongly electrophilic. The nucleophilic mechanism provides the simplest explanation for the sensitization properties of these compounds. We did not find any information about the reaction chemistry of compound **11**.

Without quantitative relative reactivity data for these sulfur nucleophiles, looking for an overall structure–potency relationship would be premature.

The potency of many electrophilic sensitizers is correlated not only with their reactivity but also with their hydrophobicity. Hydrophobicity is quantified by logP, the logarithm of the octanol/water partition coefficient. There are various methods available for experimental determination of logP and several methods for calculating it from the structure. Here, except where otherwise stated, logP values are calculated by Bio-Loom for Windows v. 1.6, BioByte Corp., Claremont, CA, USA, and are referred to as ClogP. It is noteworthy that the most potent sensitizer in Table 1, compound **1**, is the most hydrophobic with a ClogP value of ca. 6.5 and (together with compound **13**) has the least acidic SH group. The much weaker sensitizer compound **13** should react similarly to **1** but is much less hydrophobic.

## Phosphorus Nucleophiles

We found only four phosphorus nucleophiles, **14–17**, with LLNA data. These are all triesters of phosphorus acid, as shown in Table 2. One further compound in Table 2, triethyl phosphite, **18**, has GPMT data but not LLNA data.

**Table 2:** Phosphorus Nucleophiles

name	structure	CAS no.	EC3 (%)	pEC3	ClogP
triphenyl phosphite	(PhO)_3_P **14**	101-02-0	1.4	2.34	5.73
isodecyl phosphite	(Me_2_CH(CH_2_)_7_O)_3_P **15**	25448-25-3	20	1.40	12.0
diisodecylphenyl phosphite	(Me_2_CH(CH_2_)_7_O)_2_POPh **16**	25550-98-5	41	1.03	10.09
triisotridecyl phosphite	(Me_2_CH(CH_2_)_10_O)_3_P **17**	77745-66-5	92.1	0.83	12.0
triethyl phosphite	(EtO)_3_P **18**	122-52-1	19/20 in GPMT (5% injection induction, 100% topical induction, 100% challenge)	N/A	0.51

For electrophilic skin sensitizers whose potency is logP dependent, the optimal logP value for maximum potency is around 5.5.^14,15^ Three of the compounds, **15**–**17**, are extremely hydrophobic, with ClogP values in double figures, well above the optimal value for maximum sensitization potency, and they are very weak sensitizers. Compound **14** has a ClogP value of 5.73, only slightly above the optimal value, and compound **18** has a ClogP value of 0.51. Both are strong sensitizers.

## Carbon Nucleophiles

**Figure 3:**
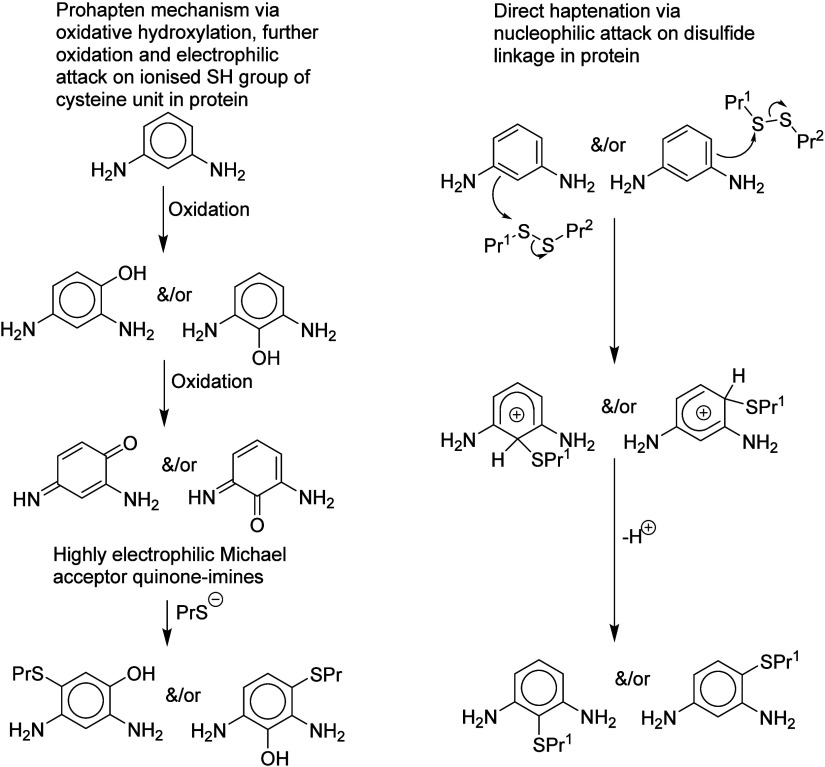
Prohapten and nucleophilic mechanisms illustrated for 1,3-diaminobenzene.

Aromatic compounds with two amino groups, two hydroxyl groups, or one of each, meta to each other, are often used as couplers in hair colorants. Although these compounds lack alerts for direct electrophilic reactivity, most of them are skin sensitizers. To rationalize their sensitization potency a pro-hapten mechanism has previously been suggested,^16^ involving activation by oxidation to introduce a hydroxyl group ortho or para to the amino or hydroxyl groups already present; subsequent further oxidation would produce a highly electrophilic quinone, diimine, or quinone–imine (Figure 3). However, since their role in hair colorants is to act as carbon-centered nucleophiles reacting with electrophilic quinone–imines or diimines, a nucleophilic mechanism for sensitization seems at least equally plausible, as shown in Figure 3.

**Figure 4:**
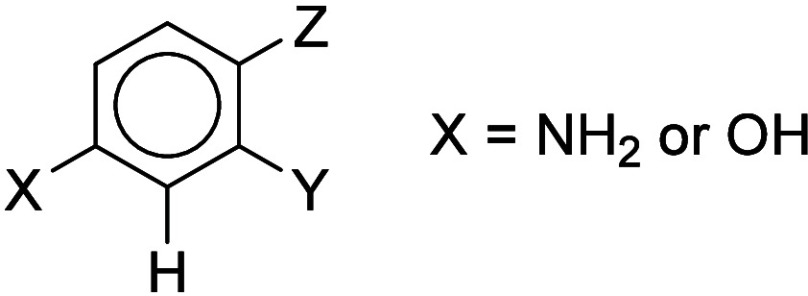
General structure of carbon-centered nucleophiles used to derive a QMM.

Table 3 lists the cases where a carbon-centered nucleophilic mechanism provides the simplest explanation for sensitization in the LLNA.

**Table 3:**
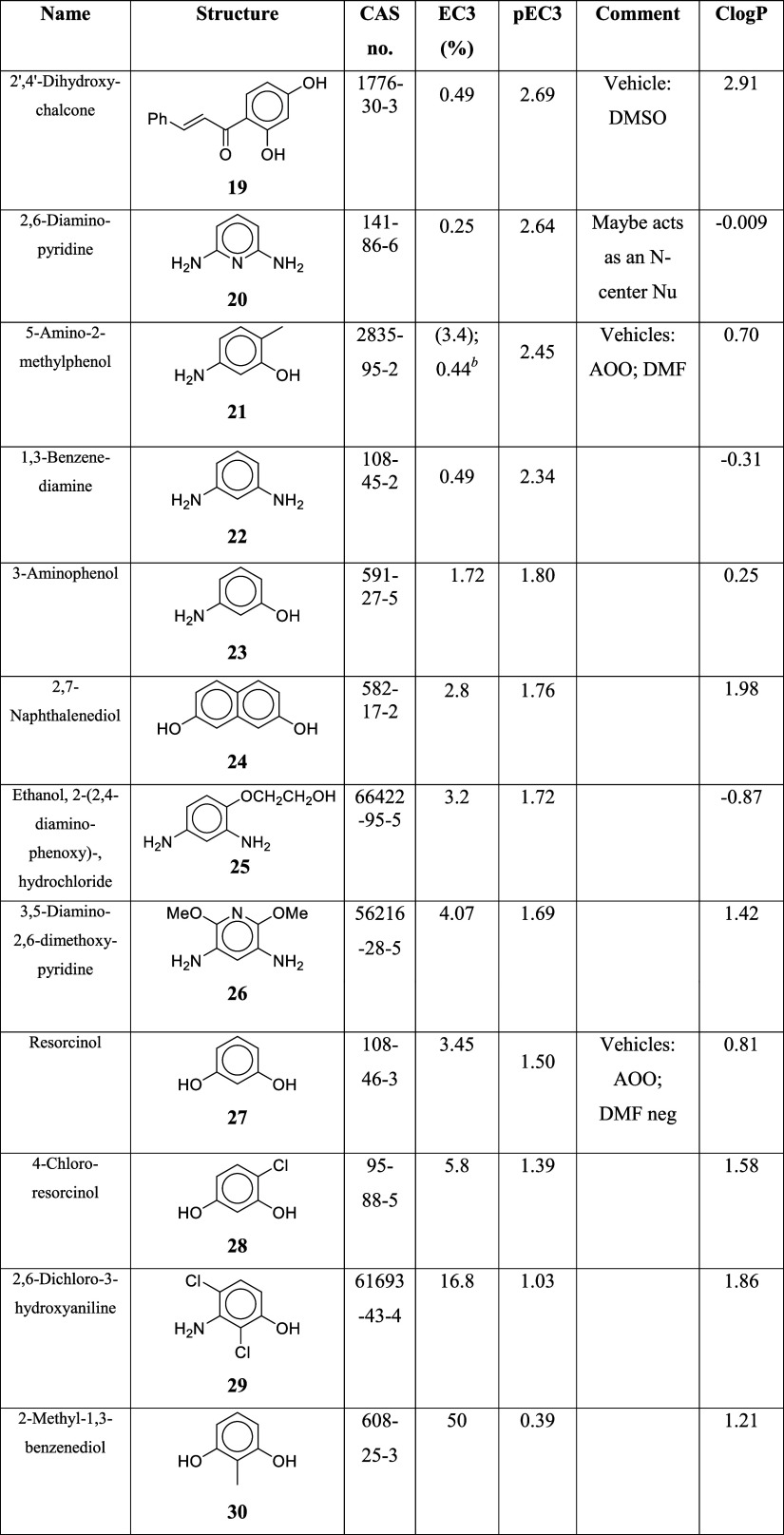
Carbon-Centered Nucleophiles^a^

a The compounds are listed in decreasing order of molecular potency (pEC3 values). The LLNA vehicle is acetone–olive oil (AOO) unless otherwise indicated.

b The EC3 value of 3.4 is based on an LLNA study using dimethyl formamide (DMF) as the vehicle. With acetone/olive oil (AOO) as the vehicle, the EC3 value was 0.44. For the QMM analysis described below, the AOO EC3 value of 0.44 was used, since the EC3 values of most of the other compounds were based on AOO as vehicle (the only exception being **19**, tested in dimethyl sulfoxide (DMSO)).

### Toward a Quantitative Mechanistic Model (QMM)

Although experimental reactivity data are unavailable for the chemicals in Table 3, Hammett substituent constants can be applied as reactivity parameters to look for a quantitative relationship between the structure and the potency. Seven of the chemicals have the common structural feature of a single benzene ring with two activating groups meta to each other and an unsubstituted carbon atom between them (Figure 4), and these were therefore chosen for a correlation study. Nucleophilic reactivity should mainly be influenced by the π-electron-donating effects of the X and Y groups and the electronic effects of any other substituents present in the ring. The special Hammett constants, σ^+^, are appropriate for modeling reactions involving π-electron-donating effects. Compilations of Hammett σ^+^ constants are available but only for para substituents. For the present purposes, ortho σ^+^ constants would be most appropriate, but since these are not available, it is assumed, as a simplifying approximation, that ortho-σ^+^ can be represented by para-σ^+^. The effects of ortho substituents are in many cases, but not all, quite well modeled by para substituent constants.^17^ The standard Hammett σ constants are used for the various meta substituents, *Z*. Here, σ^+^ and σ(meta) constants are taken from a compilation by Hansch et al.^18^

Table 4 shows the σ^+^ values used in this analysis. Based on these, the reactivity parameter Σσ^+^ is calculated for each compound by summing the σ^+^ values for all substituents. Table 5 shows the seven compounds with their Σσ^+^ values, ClogP values, and LLNA potency values pEC3 (negative log of the %EC3 value after division by the molecular weight).

**Table 4:** Hammett σ^+^ Values

substituent	σ^+^	comment
*ortho*-OH	–0.92	based on σ^+^(para)
*meta*-OH	+0.13	based on σ(meta)
*ortho*-NH_2_	–1.30	based on σ^+^(para)
*ortho*-O^–^	–2.30	based on σ^+^(para)
*meta*-Cl	+0.37	based on σ(meta)
*meta*-Me	–0.06	based on σ (meta)
*meta*-CO·CH=CHPh	+0.36	based on σ(meta) COPh
*meta*-OCH_2_CH_2_OH	+0.10	based on σ(meta) OEt
*ortho*-OMe	–0.81	based on σ^+^(para) OEt
*meta*-OMe	+0.12	based on σ(meta) OEt
ring N in pyridines, meta	+0.73	based on σ(meta)
ring N in pyridines, para	+0.83	based on σ(para)
*meta*-Ph	+0.05	based on σ(meta)
*ortho*-Ph	–0.18	based on σ^+^(para)

**Table 5:**
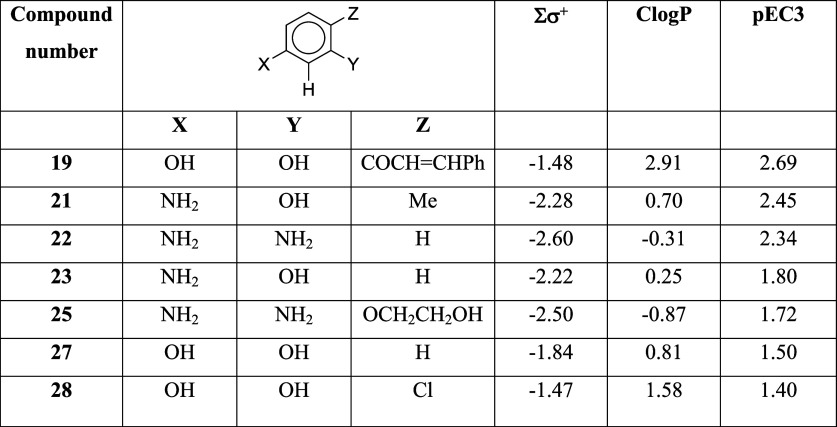
Reactivity and Hydrophobicity Parameters

Inspection of the data in Table 5 suggests that potency is dependent upon reactivity and hydrophobicity. 1,3-Benzenediamine (**22**) is more reactive (based on its Σσ^+^ value) and more potent than compound **23 (**3-aminophenol), which is a more reactive and more potent sensitizer than resorcinol (**27**). Compound **19**, 2′,4′-dihydroxychalcone, although it is not the most reactive, is the most potent. It is more hydrophobic, by more than 1 logP unit, than any of the other compounds. With only seven compounds in the data set, a multiple regression approach would not be appropriate, so to evaluate the possible dependence of potency on reactivity and hydrophobicity, we combined Σσ^+^ and logP together in the composite parameter relative alkylation index (RAI),^19^ calculated as RAI = −Σσ^+^ + 0.4 logP. Our choice of 0.4 as the logP coefficient is based on previous findings that in earlier skin sensitization QMMs based on a combination of a reactivity parameter and logP the relative contribution of the logP parameter is about 0.4 times that of the reactivity parameter.^20,21^

**Figure 5:**
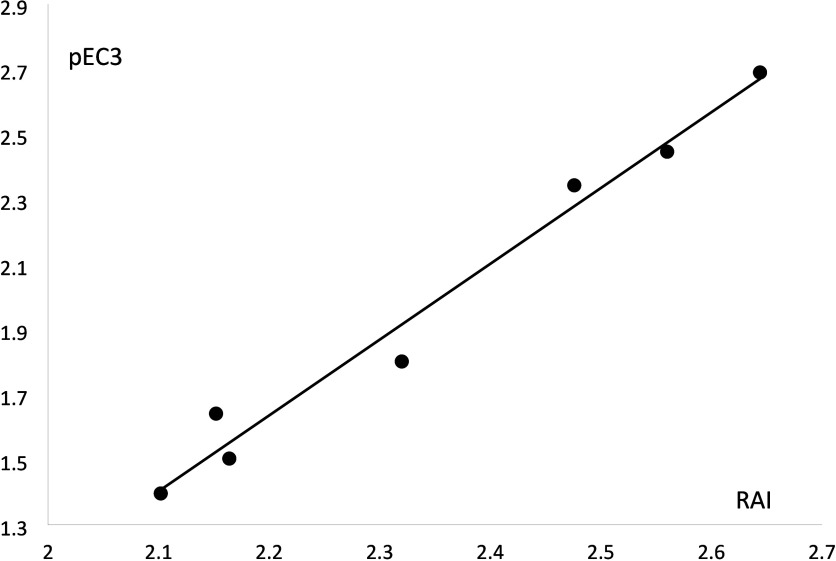
pEC3 vs RAI for C-centered nucleophilic sensitizers.

A plot of pEC3 vs RAI for the seven compounds in Table 5 is shown in Figure 5 and gives the equation
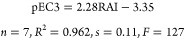
1

Equation 1 is based on several simplifying assumptions and approximations. We do not regard it as a predictive tool for risk assessment purposes. However, we consider the model applicable as a starting point for further exploration of structure–potency trends in the nucleophilic sensitization domain. Below we apply eq 1 to consider some examples and trends that have previously been difficult to rationalize in terms of electrophilic sensitization mechanisms. It needs to be taken into account that, as has been observed for electrophilic sensitizers, above a logP value of about 5.5, the dependence of potency on hydrophobicity becomes negative.^14,20^ Since the rationale for this^14^ is based on physical chemistry rather than on reaction chemistry, it is reasonable to assume that this reversal of logP dependency should apply to all reaction mechanistic domains, including the nucleophilic sensitization domain. We note that a logP limit of about 5.5 also applies in general narcosis QSARs for fish toxicity: up to this value, plots of toxicity vs logP are linear, but they flatten out as logP increases further.^22,23^ To enable predictive extrapolation of eq 1 for compounds with logP values above 5.5, an adjusted logP value, logP_adj_, can be used, whereby the amount by which logP exceeds 5.5 is subtracted from 5.5 to give logP_adj_. Thus, for compounds with logP > 5.5, logP_adj_ = 11 – logP. We can now consider the compounds in Table 3 that were not used in the derivation of eq 1.

### Compounds **20**, 2,6-Diaminopyridine, and **26**, 3,5-Diamino-2,6-dimethoxypyridine

**Figure 6:**
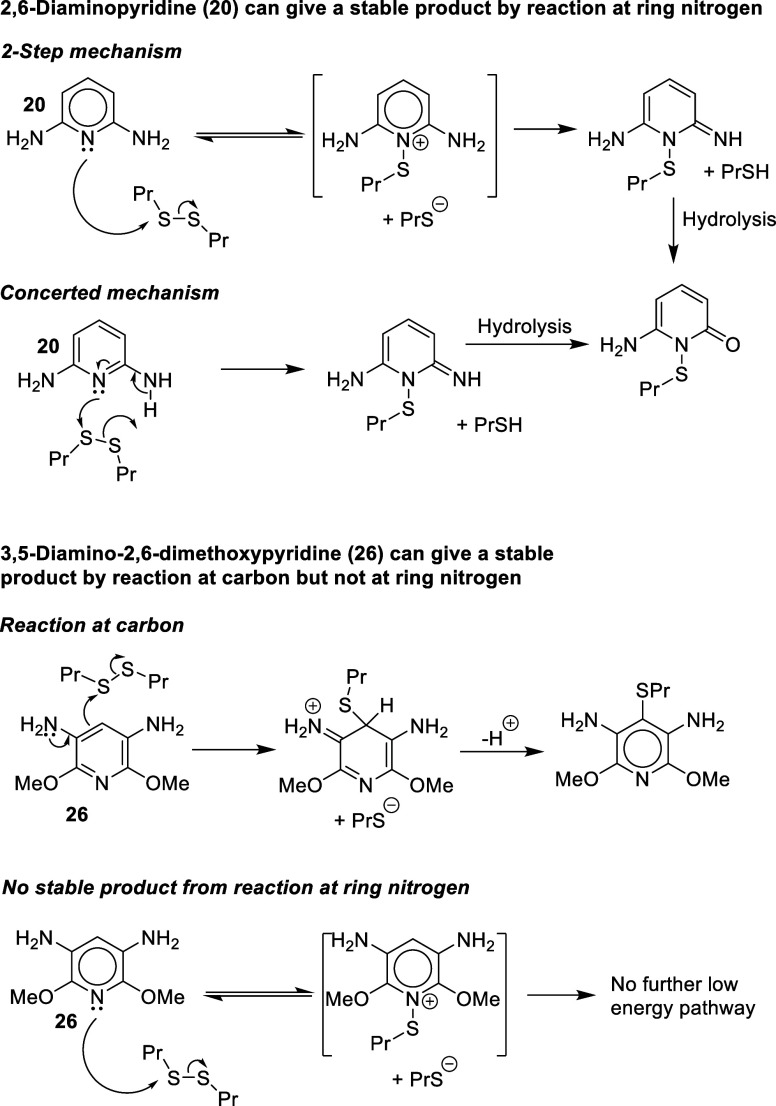
2,6-Diaminopyridine (**20**), and 3,5-diamino-2,6-dimethoxypyridine (**26**).

In compound **20**, the ring nitrogen is the most likely reaction site. Having a quaternary nitrogen bonded to a divalent sulfur, the initial adduct can lose a proton from one of the amino groups to give a stable uncharged adduct (Figure 6). In compound **26**, the initial adduct from the reaction at the ring nitrogen cannot form a stable derivative by the loss of a proton. However, it can react at the carbon atom para to the nitrogen, acting as a carbon-centered nucleophile analogous to 1,3-diamino benzene (**22**) but with deactivation effects from the electronegative ring nitrogen in the para position and the two electronegative methoxy groups in the meta positions. These deactivation effects are represented by the positive Hammett constants used to calculate Σσ^+^ for this compound:
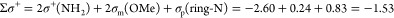
Combining this Σσ^+^ value with the ClogP value of 1.42 to obtain an RAI value of 2.10 and using this RAI value in eq 1 gives a calculated EC3 value of 6.2%, in agreement with the weaker sensitization potency of compound **26** compared to compound **22** and not greatly different (within 95% confidence limits of eq 1) from the experimental value of 4.07%.

### Compound **24**, 2,7-Dihydroxynaphthalene

**Figure 7:**
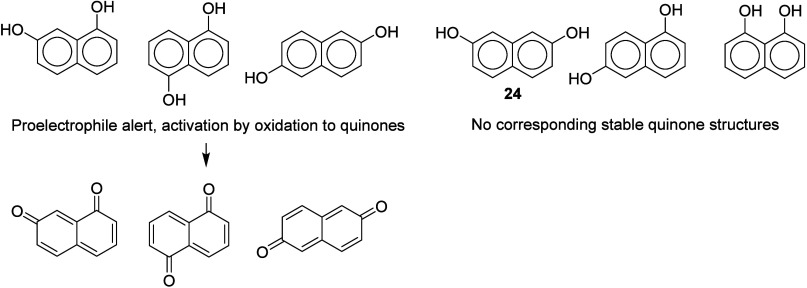
Dihydroxynaphthalenes.

There are six dihydroxynaphthalenes with one −OH group in each ring (Figure 7). Three of these can be oxidized to highly electrophilic (Michael acceptor) quinone-type derivatives and would be predicted to be strong sensitizers acting via this mechanism. The other three, compound **24** being one of them, cannot form quinone-type structures. However, sensitization by a nucleophilic mechanism may be possible. For compound **24**, the potential reaction sites C1 and C3 experience the electronic effects of the *ortho*-OH in the same ring and the OH group in the other ring plus the impact of the aromatic ring fused to the ring where the reaction occurs. These effects can be modeled for the original Hammett constants σ as follows.^24^

For reaction at C1:

Effect of substituent at C7 = 0.35(σ_meta_ + σ_para_)
Effect of fused aromatic ring = σ_meta-Ph_ + σ_ortho-Ph_

For reaction at C3:

Effect of substituent at C7 = 0.13σ_meta_ + 0.41σ_para_
Effect of fused aromatic ring = σ_meta-Ph_ + σ_ortho-Ph_

Assuming these relationships also apply to σ^+^ values, the Σσ^+^ values for reactions at C1 and at C3 can be calculated as follows.

Reaction at C1:

Σσ^+^ = Effects of [2-OH (−0.92) + 7-OH (=0.35(−0.92 + 0.13)) + fused ring (=0.05–0.18)] = −1.33

Reaction at C3:

Σσ^+^ = Effects of [2-OH (−0.92) + 7-OH (=0.13 × 0.13–0.41 × 0.92)) + fused ring (=0.05–0.18)] = −1.41

Based on the Σσ^+^ values, 2,7-dihydroxynaphthalene is predicted to be more reactive at the C3 position than at the C1 position.

Combining the Σσ^+^ value for the reaction at C1 with the ClogP value and applying eq 1 gives a calculated EC3 value of 3.4%. The reported experimental value of 2.8% is within the 95% confidence limits of eq 1.

### 2,6-Dichloro-3-hydroxyaniline, **29**, and 2-Methyl Resorcinol, **30**

In these compounds, the position ortho to both activating groups is blocked so the reaction can only occur at a position ortho to one activating group and para to the other (Table 6). Based on the assumption that σ^+^(ortho) = σ^+^(para), eq 1 gives predicted EC3 values substantially lower than the reported EC3 values, i.e., it overpredicts the potency, as shown in Table 6. There are two complementary interpretations of this. First, it is likely that the true σ^+^(ortho) values are more negative (indicating higher reactivity) than the corresponding σ^+^(para). If the difference between σ^+^(ortho) and σ^+^(para) does not vary greatly, the σ^+^(para) values can still be used in a model such as eq 1 for a set of chemicals where the reaction center is ortho to both activating groups because the difference is in effect corrected for in the RAI coefficient. However, when eq 1 is applied to a compound with one activating group para to the reaction center, the RAI coefficient effectively applies a false correction for the para activating group, leading to overprediction. Second, with a substituent between the two activating groups, steric effects can prevent the activating substituents from aligning in the plane of the aromatic ring, reducing the π bonding of the activating group electrons with the aromatic ring. A methyl group has a more significant steric effect than a chloro substituent,^25^ consistent with eq 1 overpredicting compound **30** to a greater extent than it overpredicts compound **29**.

**Table 6:**
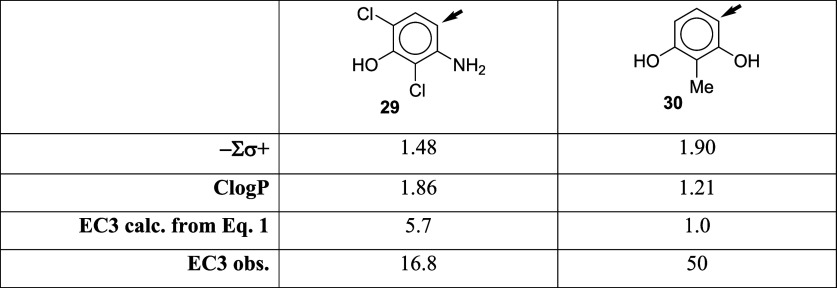
Application of Equation 1 to 2,6-Dichloro-3-hydroxyaniline, **29**, and 2-Methyl Resorcinol, **30**^a^

a The reaction center is indicated by the arrow.

### Carbon-Centered Nucleophiles with a Single Activating Group

Numerous phenols and aromatic monoamines have been reported as skin sensitizers despite not having electrophilicity alerts. Below we discuss some of the cases we are aware of in light of eq 1.

### Anacardic Acid, Cardanol, and Cardol

These three chemicals, shown in Table 7, are the major constituents of the liquid from the shell of the cashew nut (*Anacardium occidentale*),^26^ with the R group predominantly a mixture of unbranched C15 saturated and mono-, di-, and triunsaturated chains. They are also found in other plants, notably *Ginkgo biloba*, in which the chain lengths cover a wider range (at least C13–C19) and can include tetraunsaturated components.^27^ Cashew nut shell liquid (CNSL) in the original state is mainly composed of anacardic acid with lower levels of cardanol and cardol. Although cardanol is typically present in CNSL at only about 5%, it is readily formed from anacardic acid by decarboxylation and is a major industrial raw material. The relative proportions of components in CNSL vary between sources and depending on the extraction process conditions. Based on guinea pig and clinical evidence, anacardic acid and cardol are strong sensitizers.^28−31^ Cardanol is reported to have some sensitization potency but is much weaker than cardol and anacardic acid.^27,28^ None of these chemicals have electrophilicity or pro-electrophilicity alerts. We now consider them from the perspective of the nucleophilic sensitization mechanism.

**Table 7:**
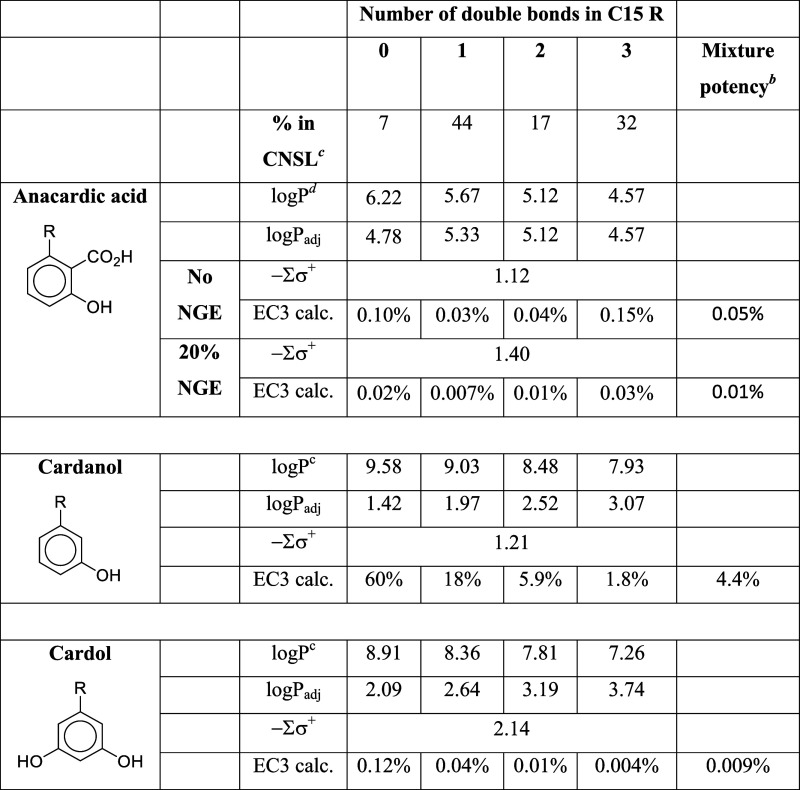
Anacardic Acid, Cardanol, and Cardol^a^

a For anacardic acid, which would be mainly ionized at physiological pH, the logP value is based on the carboxylate ion. The σ(meta) value of 0.09 given by Hansch et al.^18^ is used for the ionized CO_2_H group. For the R group, a σ^+^ value of −0.29, based on the value given by Hansch et al.^18^ for *n*-butyl, is used.

b These figures are calculated by addition of toxic units: 100/EC3_mix_ = %_A_/EC3_A_ + %_B_/EC3_B_...

c Rounded average of figures given by Symes and Dawson^32^ and Caillol.^33^

d LogP values calculated manually by the method of Leo and Hansch.^34^ Details of these calculations are given in SI3.

**Table 8:**
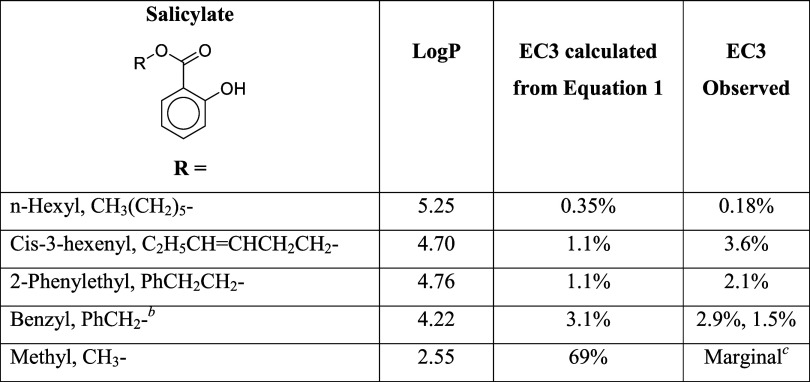
LLNA Data for Salicylate Esters^a^

a Observed EC3 values are taken from Belsito et al.^38^ LogP values are calculated manually by the method of Leo and Hansch^34^ starting from the experimental value of 2.55 for methyl salicylate^39^ and agree within 0.2 log units with published values calculated by various computer methods.

b For benzyl salicylate, an alternative electrophilic mechanism involving S_N_2 attack at the benzyl carbon, with the salicylate anion acting as the leaving group, is also possible. Benzyl benzoate is also proposed to sensitize via this mechanism^40^ and has an EC3 value of 17%. Benzyl salicylate would be predicted to be a stronger sensitizer than benzyl benzoate if they both act as S_N_2 electrophilic sensitizers. This is because the salicylate ion should be a better leaving group than the benzoate ion, since it is less basic (p*K*_a_ values of benzoic acid and salicylic acid are 4.20 and 2.97, respectively^41^).

c Belsito et al.^38^ summarize four LLNA assays on methyl salicylate with acetone/olive oil as the vehicle, all negative at concentrations up to 25%, and one test in dimethyl formamide giving an EC3 value of 25%.

**Figure 8:**
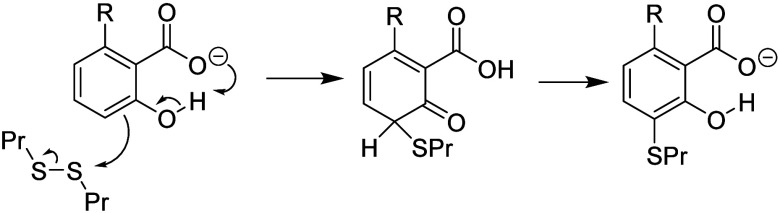
Neighboring group effect for anacardic acid.

Clearly cardol, having two activating OH groups and a −Σσ^+^ value of 2.14, is expected to be more reactive than cardanol (−Σσ^+^ = 1.21). These −Σσ^+^ values apply for all chain lengths and degrees of unsaturation of the R group. At first sight, anacardic acid might be expected to be slightly less reactive than cardanol, having a −Σσ^+^ value of 1.12. However, this −Σσ^+^ value does not consider the neighboring group effect (NGE) of the ionized carboxylate group (Figure 8). This makes the phenolic OH group (σ^+^ = −0.92) more like an ionized phenol group (σ^+^ = −2.30), so the nucleophilic reactivity would be better modeled by a more negative Σσ^+^ value, somewhere between −1.12 and −2.5. We have carried out calculations based on no NGE (Σσ^+^ value of −1.12) and a 20% NGE (Σσ^+^ value of −1.40, based on 0.20 σ^+^(O^–^) + 0.80 σ^+^(OH)).

Equation 1 correctly predicts the high potency of cardol and anacardic acid and correctly identifies cardanol as being much less potent.

## Salicylate Esters

Hexyl salicylate is a strong sensitizer in the LLNA (EC3 = 0.18%) but is a very weak sensitizer in humans, with a Human Sensitization Potency Category of 4.^35^ It is often considered as an LLNA false positive (e.g., Natsch et al.^36^), but given clinical reports of human skin allergy not only to hexyl salicylate but also to other salicylate esters^37^ together with generally weak positive results in guinea pig studies for several salicylates,^38^ it is better described as a true sensitizer whose human potency is substantially overestimated by the LLNA. Several other salicylate esters have been tested in the LLNA: these together with hexyl salicylate are shown in Table 8 with the predictions of eq 1 and their reported EC3 values. The Σσ^+^ value for all of these salicylates is −0.60.

Overall, the LLNA potency of salicylate esters is quite well modeled by the nucleophilic sensitization mechanism. The problematic question is no longer “Why is hexyl salicylate such a strong LLNA positive?” but “Why are salicylate esters not much more potent in humans?”.

## Conclusions

Nucleophilic sensitizers constitute a “new” reaction mechanistic applicability domain. Although the concept of nucleophilic sensitization is not new, it is now apparent that it is not such a rare mechanism as had previously been thought.^3^ The QMM developed here based on σ^+^ and logP provides a useful starting point for further exploration of the nucleophilic sensitization domain, as illustrated by our analysis of the data on anacardic acids, cardols, and cardanols, but being based on only seven compounds, it is currently of limited predictive value. To develop a more generally applicable predictive capability, an experimental method for determining nucleophilic reactivity toward electrophilic disulfides would be desirable. One possibility would be to use 4-nitrothiophenolate, which is easily analyzed spectroscopically^42^ as the leaving group in a model substrate such as CH_3_SSC_6_H_4_NO_2_(*p*).

The nucleophilic sensitization domain has not so far been recognized in formally validated defined approaches for nonanimal-based detection of skin sensitization potential and potency, which are based primarily on the premise that skin sensitization is based on reactions of electrophiles with skin protein-based nucleophiles.

In particular, peptide reactivity assays such as DPRA and kDPRA would not be expected to detect nucleophilic sensitizers. This is because the peptides used are nucleophilic and do not contain disulfide linkages that could react with nucleophilic sensitizers. The KeratinoSens assay is in effect an assay for electrophilic reactivity to thiols since it depends on covalent modification of thiol groups on Keap proteins.^43^

If Keap thiol modification is the only trigger for a positive response, the KeratinoSens assay would not be expected to detect nucleophilic sensitizers. Other cell-based assays, such as h-CLAT and GARD, that rely less specifically on modification of a particular protein are more likely to detect nucleophilic sensitizers, as are also reconstituted skin assays.

In work currently ongoing, we intend to follow up the present paper with an assessment of the performance of current nonanimal assays for nucleophilic sensitizers and to consider how mechanism-based in silico models can be extended to cover this mechanistic domain.

## Supporting Information Available

CAS numbers and SMILES representations for compounds listed in Tables 1–3; source references for LLNA data listed in Tables 1-3; manual calculation of logP for anacardic acid, cardanol, and cardol (PDF)

## References

[ref1] Chilton M. L.; Api A. M.; Foster R. S.; Gerberick G. F.; Lavelle M.; Macmillan D. S.; Na M.; O’Brien D.; O’Leary-Steele C.; Patel M.; Ponting D. J.; Roberts D. W.; Safford R. J.; Tennant R. E. Updating the Dermal Sensitisation Thresholds Using an Expanded Dataset and an in Silico Expert System. Regul. Toxicol. Pharmacol. 2022, 133, 105200 10.1016/j.yrtph.2022.105200. 35662638

[ref2] Roberts D. W.; Lepoittevin J.-P. Hapten-Protein Interactions. Allergic Contact Dermatitis; Springer Berlin Heidelberg: Berlin, Heidelberg, 1998; pp 81–111; 10.1007/978-3-642-80331-4_6.

[ref3] Roberts D. W.; Basketter D.; Kimber I.; White J.; McFadden J.; White I. R. Sodium Metabisulfite as a Contact Allergen - An Example of a Rare Chemical Mechanism for Protein Modification. Contact Dermatitis 2012, 66 (3), 123 10.1111/j.1600-0536.2011.02038.x. 22320665

[ref4] Drewe W. C.; Payne M. P.; Williams R. V. Phosphite Esters: A Novel Class of Contact Allergen. Contact Dermatitis 2017, 76 (5), 312 10.1111/cod.12704. 28386969

[ref5] Kortemme T; Creighton T. E. Ionisation of Cysteine Residues at the Termini of Model Alpha-helical Peptides. Relevance to Unusual Thiol pKa Values in Proteins of the Thioredoxin Family. J. Mol. Biol. 1995, 253 (5), 799 10.1006/jmbi.1995.0592. 7473753

[ref6] Schneider J. F.; Westley J. Metabolic Interrelations of Sulfur in Proteins, Thiosulfate, and Cystine. J. Biol. Chem. 1969, 244 (20), 5735 10.1016/S0021-9258(18)63621-X. 5348607

[ref7] Harvey R. G.; Jacobson H. I.; Jensen E. V. Phosphonic Acids. VI. The Reaction of Trivalent Phosphorus Esters with Organic Disulfides. J. Am. Chem. Soc. 1963, 85 (11), 1618 10.1021/ja00894a018.

[ref8] Chipinda I.; Hettick J. M.; Simoyi R. H.; Siegel P. D. Oxidation of 2-Mercaptobenzothiazole in Latex Gloves and Its Possible Haptenation Pathway. Chem. Res. Toxicol. 2007, 20 (8), 1084 10.1021/tx700139g. 17630704

[ref9] Malouki M. A.; Richard C.; Zertal A. Photolysis of 2-Mercaptobenzothiazole in Aqueous Medium Laboratory and Field Experiments. J. Photochem. Photobiol. A Chem. 2004, 167 (2–3), 121 10.1016/j.jphotochem.2004.04.010.

[ref10] Chipinda I.; Hettick J. M.; Simoyi R. H.; Siegel P. D. Zinc Diethyldithiocarbamate Allergenicity: Potential Haptenation Mechanisms. Contact Dermatitis 2008, 59 (2), 79 10.1111/j.1600-0536.2008.01399.x. 18759874

[ref11] Tipman R. N.; Leja J. Reactivity of Xanthate and Dixanthogen in Aqueous Solutions of Different PH. Colloid Polym. Sci., Kolloid Z. Z. Polym. 1975, 253 (1), 4 10.1007/BF01419251.

[ref12] Jones M. H.; Woodcock J. T. Decomposition of Alkyl Dixanthogens in Aqueous Solutions. Int. J. Miner. Process. 1983, 10 (1), 1 10.1016/0301-7516(83)90030-3.

[ref13] Zard S. Z. On the Trail of Xanthates: Some New Chemistry from an Old Functional Group. Angewandte Chemie (International Edition in English). 1997, 36, 672 10.1002/anie.199706721.

[ref14] Roberts D. W.; Basketter D. A. Quantitative Structure-Activity Relationships: Sulfonate Esters in the Local Lymph Node Assay. Contact Dermatitis 2000, 42 (3), 154 10.1034/j.1600-0536.2000.042003154.x. 10727166

[ref15] Roberts D. W.; Patlewicz G.; Kern P. S.; Gerberick F.; Kimber I.; Dearman R. J.; Ryan C. A.; Basketter D. A.; Aptula A. O. Mechanistic Applicability Domain Classification of a Local Lymph Node Assay Dataset for Skin Sensitization. Chem. Res. Toxicol. 2007, 20 (7), 1019 10.1021/tx700024w. 17555332

[ref16] Aptula A. O.; Enoch S. J.; Roberts D. W. Chemical Mechanisms for Skin Sensitization by Aromatic Compounds with Hydroxy and Amino Groups. Chem. Res. Toxicol. 2009, 22 (9), 1541 10.1021/tx9000336. 19678610

[ref17] Charton M. Nature of the Ortho Effect. V. ortho-Substituent Constants. J. Am. Chem. Soc. 1969, 91, 6649 10.1021/ja01052a020.

[ref18] Hansch C.; Leo A.; Taft R. W. A Survey of Hammett Substituent Constants and Resonance and Field Parameters. Chem. Rev. 1991, 91 (2), 165 10.1021/cr00002a004.

[ref19] Roberts D. W.; Williams D. L. The Derivation of Quantitative Correlations Between Skin Sensitization and Physicochemical Parameters for Alkylating Agents and their Application to Experimental Data for Sultones. J. Theor. Biol. 1982, 99, 807 10.1016/0022-5193(82)90199-0. 6191155

[ref20] Roberts D. W.; Aptula A. O.; Patlewicz G. Mechanistic Applicability Domains for Non-Animal Based Prediction of Toxicological Endpoints. QSAR Analysis of the Schiff Base Applicability Domain for Skin Sensitization. Chem. Res. Toxicol. 2006, 19 (9), 1228 10.1021/tx060102o. 16978028

[ref21] Roberts D. W.; Aptula A.; Api A. M. Structure-Potency Relationships for Epoxides in Allergic Contact Dermatitis. Chem. Res. Toxicol. 2017, 30 (2), 524 10.1021/acs.chemrestox.6b00241. 28121139

[ref22] Könemann H. Quantitative Structure-Activity Relationships in Fish Toxicity Studies Part 1: Relationship for 50 Industrial Pollutants. Toxicology 1981, 19 (3), 209 10.1016/0300-483X(81)90130-X. 7233445

[ref23] Veith G. D.; Call D. J.; Brooke L. T. Structure - Toxicity Relationships for the Fathead Minnow, Pimephales Promelas: Narcotic Industrial Chemicals. Can. J. Fish. Aquat. Sci. 1983, 40 (6), 743 10.1139/f83-096.

[ref24] Perrin D. D.; Dempsey B.; Serjeant E. P. pKa Prediction for Organic Acids and Bases; Chapman and Hall: London, 1981.

[ref25] Taft R. W. Polar and Steric Substituent Constants for Aliphatic and ο-Benzoate Groups from Rates of Esterification and Hydrolysis of Esters. J. Am. Chem. Soc. 1952, 74 (12), 3120 10.1021/ja01132a049.

[ref26] Hemshekhar M.; Sebastin Santhosh M.; Kemparaju K.; Girish K. S. Emerging Roles of Anacardic Acid and Its Derivatives: A Pharmacological Overview. Basic and Clinical Pharmacology and Toxicology. 2012, 110, 122 10.1111/j.1742-7843.2011.00833.x. 22103711

[ref27] Lepoittevin J. P.; Benezra C.; Asakawa Y. Allergic Contact Dermatitis to Ginkgo Biloba L.: Relationship with Urushiol. Arch. Dermatol. Res. 1989, 281 (4), 227 10.1007/BF00431055. 2774654

[ref28] Schmidt R. J.; Khan L.; Chung L. Y. Are Free Radicals and Not Quinones the Haptenic Species Derived from Urushiols and Other Contact Allergenic Mono- and Dihydric Alkylbenzenes? The Significance of NADH, Glutathione, and Redox Cycling in the Skin. Arch. Dermatol. Res. 1990, 282 (1), 56 10.1007/BF00505646. 2317084

[ref29] Rozas-Muñoz E.; Gamé D.; Mir-Bonafé J. F.; Piquero-Casals J. Plant Contact Dermatitis in 2021. Current Treatment Options in Allergy. 2022, 9, 76 10.1007/s40521-022-00303-8.

[ref30] Marks J. G.; DeMelfi T.; McCarthy M. A.; Witte E. J.; Castagnoli N.; Epstein W. L.; Aber R. C. Dermatitis from Cashew Nuts. J. Am. Acad. Dermatol. 1984, 10 (4), 627 10.1016/S0190-9622(84)80269-8. 6715612

[ref31] Baer H.; Watkins R. C.; Bowser R. T. Delayed Contact Sensitivity to Catechols and Resorcinols. The Relationship of Structure and Immunization Procedure to Sensitizing Capacity. Immunochemistry 1966, 3 (6), 479 10.1016/0019-2791(66)90133-9.

[ref32] Symes W. F.; Dawson C. R. Cashew Nut Shell Liquid. IX. The Chromatographic Separation and Structural Investigation of the Olefinic Components of Methylcardanol. J. Am. Chem. Soc. 1953, 75 (20), 4952 10.1021/ja01116a021.

[ref33] Caillol S. Cardanol: A Promising Building Block for Biobased Polymers and Additives. Current Opinion in Green and Sustainable Chemistry. 2018, 14, 26 10.1016/j.cogsc.2018.05.002.

[ref34] Hansch C.; Leo A. J. Substituent Constants for Correlation Analysis in Chemistry and Biology; Wiley and Sons: New York, 1979. 10.1021/jm00212a024 836503

[ref35] Basketter D. A.; Alépée N.; Ashikaga T.; Barroso J.; Gilmour N.; Goebel C.; Hibatallah J.; Hoffmann S.; Kern P.; Martinozzi-Teissier S.; Maxwell G.; Reisinger K.; Sakaguchi H.; Schepky A.; Tailhardat M.; Templier M. Categorization of Chemicals According to Their Relative Human Skin Sensitizing Potency. Dermatitis. 2014, 25, 11 10.1097/DER.0000000000000003. 24407057

[ref36] Natsch A.; Kleinstreuer N.; Asturiol D. Reduced Specificity for the Local Lymph Node Assay for Lipophilic Chemicals: Implications for the Validation of New Approach Methods for Skin Sensitization. Regul. Toxicol. Pharmacol. 2023, 138, 105333 10.1016/j.yrtph.2023.105333. 36608925 PMC9941753

[ref37] Mortz C. G.; Thormann H.; Goossens A.; Andersen K. E. Allergic Contact Dermatitis from Ethylhexyl Salicylate and Other Salicylates. Dermatitis 2010, 21 (2), E7 10.2310/6620.2010.09090. 20233542

[ref38] Belsito D.; Bickers D.; Bruze M.; Calow P.; Greim H.; Hanifin J. M.; Rogers A. E.; Saurat J. H.; Sipes I. G.; Tagami H. A Toxicologic and Dermatologic Assessment of Salicylates When Used as Fragrance Ingredients. Food Chem. Toxicol. 2007, 45 (1), S318 10.1016/j.fct.2007.09.066. 18022746

[ref39] Hansch C.; Leo A. The Log P Database; Pomona College: Claremont, CA, 1987. Cited in https://pubchem.ncbi.nlm.nih.gov/compound/Methyl-Salicylate.

[ref40] Roberts D. W.; Kimber I.; Basketter D. A. Specificity of the Local Lymph Node Assay (LLNA) for Skin Sensitisation. Reg. Toxicol. Pharm. 2023, 141, 105402 10.1016/j.yrtph.2023.105402. 37116738

[ref41] Smith R. M.; Martell A. E. Critical Stability Constants; Springer US: Boston, MA, 1976; Vols. 1–4; 10.1007/978-1-4757-5506-0.

[ref42] Hupe D. J.; Jencks W. P. Nonlinear Structure-Reactivity Correlations. Acyl Transfer between Sulfur and Oxygen Nucleophiles. J. Am. Chem. Soc. 1977, 99 (2), 451 10.1021/ja00444a023.

[ref43] Dinkova-Kostova A. T.; Massiah M. A.; Bozak R. E.; Hicks R. J.; Talalay P. Potency of Michael Reaction Acceptors as Inducers of Enzymes That Protect against Carcinogenesis Depends on Their Reactivity with Sulfhydryl Groups. Proc. Natl. Acad. Sci. U.S.A. 2001, 98 (6), 3404 10.1073/pnas.051632198. 11248091 PMC30666

