# Supplementary material for: Updating Reaction Mechanistic Domains for Skin Sensitization: 1. Nucleophilic Skin Sensitizers
Source: Chem Res Toxicol. 2024 Sep 11;37(11):1757–68. doi: 10.1021/acs.chemrestox.4c00207 (PMC11577424; doi:10.1021/acs.chemrestox.4c00207)
Supplement: Supplementary file 1 — tx4c00207_si_001.pdf [file tx4c00207_si_001.pdf]

SUPPORTING INFORMATION

UPDATING REACTION MECHANISTIC DOMAINS FOR SKIN SENSITIZATION: 1. NUCLEOPHILIC SKIN SENSITIZERS

David W Roberts*[a], Anne Marie Api[b], Aynur Aptula[c], Isabelle Lee[b] and Holger Moustakas[b]

a. School of Pharmacy and Biomolecular Sciences, Liverpool John Moores University, Byrom Street, Liverpool L3 3AF, England, United Kingdom

b. Research Institute for Fragrance Materials, Inc, 1200 MacArthur Blvd #306, Mahwah, NJ 07430

SEAC, Unilever, Colworth Science Park, Sharnbrook, Bedfordshire, MK44 1LQ, England, United Kingdom

*Corresponding author: d.w.roberts@ljmu.ac.uk

Table of contents

Pages 2-5. Supporting information 1. CAS numbers and SMILES representations for compounds listed in Tables 1, 2 and 3

Pages 6-10. Supporting information 2. Source references for LLNA data listed in Tables 1, 2 and 3.

Pages 11-12. Supporting information 3. Manual calculation of logP for cardanols, cardols and anacardic acids

Supporting information 1. CAS numbers and SMILES representations for compounds listed in Tables 1, 2 and 3

Table S1 CAS numbers and SMILES representations

Name	CAS	Smiles	Structure
1-Dodecanethiol	112-55-0	SCCCCCCCCCCCC	n-$C_{12}H_{25}SH$ **1**
Sodium diethyldithiocarbamate	148-18-5	[Na].S=C(S)N(CC)CC	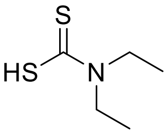 **2** (Na salt)
2-Mercaptobenzothiazole*	149-30-4	SC1=NC2=C(S1)C=CC=C2<br>S=C1SC=2C=CC=CC2N1	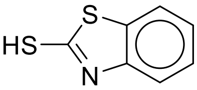 **3**<br>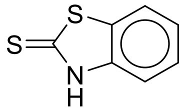 **3** (dithiocarbamate tautomer)
Glycerol monomercaptoacetate	30618-84-9	C(C(COC(=O)CS)O)O	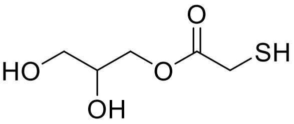 **4**
1-Thioglycerol	96-27-5	OCC(O)CS	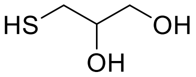 **5**
Isooctyl 3-mercaptopropionate	30374-01-7	CC(C)CCCCCOC(=O)CCS	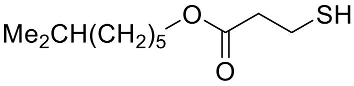 **6**
Ammonium thioglycolate	5421-46-5; 34316-71-7	O=C(O)CS.N	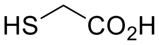 **7** (Ammonium salt)
Sodium ethyl xanthate	140-90-9	[Na].S=C(S)OCC	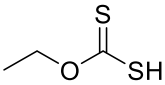 **8** (Na salt)

Carbonodithioic acid, O-(3-methylbutyl) ester, potassium salt	928-70-1	[K].S=C(S)OCCC(C)C	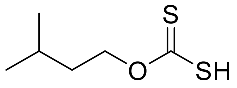 **9** (K salt)
3-Amino-1,2,4-triazole-5-thiol*	16691-43-3	NC1=NN=C(S)N1<br>S=C1NN=C(N)N1	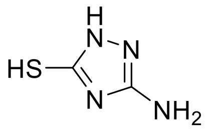 **10**<br>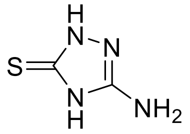 **10** (thiourea tautomer)
Sodium diisobutyldithiophosphinate	13360-78-6	[Na].S=P(S)(CC(C)C)CC(C)C	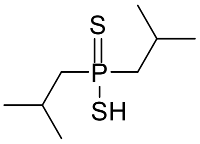 **11** (Na salt)
2-Mercaptobenzimidazole*	583-39-1	SC1=NC2=C(N1)C=CC=C2<br>S=C1NC=2C=CC=CC2N1	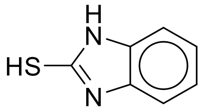 **12**<br>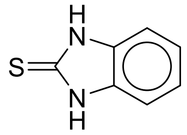 **12** (thiourea tautomer)
Isopropyl mercaptan	75-33-2	SC(C)C	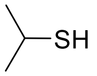 **13**
Triphenyl phosphite	101-02-0	O(C=1C=CC=CC1)P(OC=2C=CC=CC2)OC=3C=CC=CC3	$(PhO)_3P$ **14**
Isodecyl phosphite	25448-25-3	CC(C)CCCCCCCOP(OCCCCCCCC(C)C)OCCCCCCCC(C)C	$(Me_2CH(CH_2)_7O)_3P$ **15**
Diisodecylphenyl phosphite	25550-98-5	CC(C)CCCCCCCOP(OCCCCCCCC(C)C)OC1=CC=CC=C1	$(Me_2CH(CH_2)_7O)_2POPh$ **16**
Triisotridecyl phosphite	77745-66-5	CC(C)CCCCCCCCCCOP(OCCCCCCCCC	$(Me_2CH(CH_2)_{10}O)_3P$ **17**

		CC(C)C)OCCCCCCC CCCC(C)C	
Triethyl phosphite	122-52-1	O(P(OCC)OCC)CC	$(EtO)_3P$ **18**
2',4'-Dihydroxychalcone	1776-30-3	O=C(C=CC=1C=CC=CC1)C2=CC=C(O)C=C2O	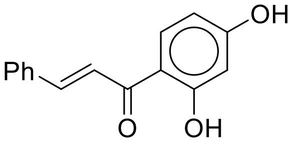 **19**
2,6-Diaminopyridine	141-86-6	N=1C(N)=CC=CC1N	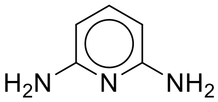 **20**
5-Amino-2-methylphenol	2835-95-2	OC1=CC(N)=CC=C1C	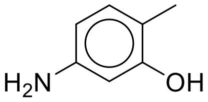 **21**
1,3-Benzenediamine	108-45-2	NC1=CC=CC(N)=C1	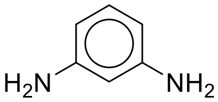 **22**
3-Aminophenol	591-27-5	OC1=CC=CC(N)=C1	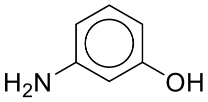 **23**
2,7-Naphthalenediol	582-17-2	OC=1C=CC2=CC=C(O)C=C2C1	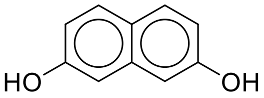 **24**
Ethanol, 2-(2,4-diaminophenoxy)-, hydrochloride	66422-95-5	Cl.OCCOC1=CC=C(N)C=C1N	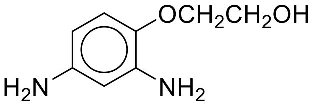 **25**
3,5-Diamino-2,6-dimethoxypyridine	56216-28-5	Cl.N=1C(OC)=C(N)C=C(N)C1OC	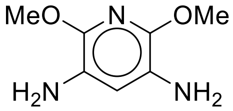 **26**
Resorcinol	108-46-3	OC1=CC=CC(O)=C1	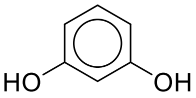 **27**
4-Chlororesorcinol	95-88-5	ClC1=CC=C(O)C=C1O	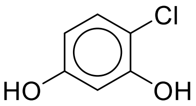 **28**

2,6-Dichloro-3-hydroxyaniline	61693-43-4	Cl.ClC1=CC=C(O)C(Cl)=C1N	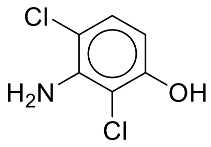 **29**
2-Methyl-1,3-benzenediol	608-25-3	OC1=CC=CC(O)=C1C	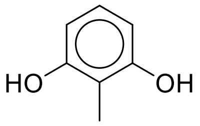 **30**

*Possibility of nucleophilic reaction via either tautomer for compounds **3**, **10** and **12**, illustrated by **3**

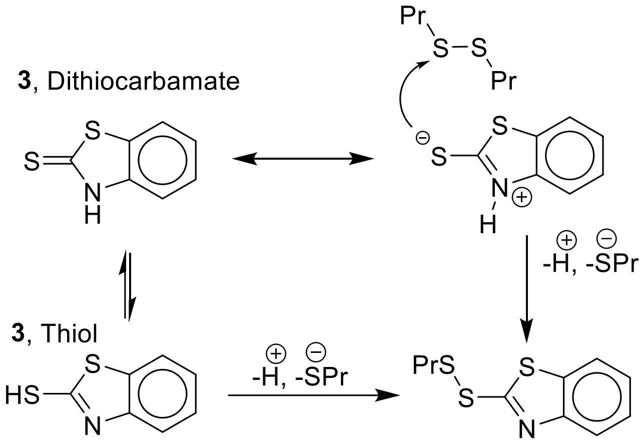

Supporting information 2. Source references for LLNA data in Tables 1-3.

For chemicals with multiple references, the EC3 value shown is the median of the individual LLNA studies.

Table S2 Sulphur nucleophiles

Structure	CAS no.	EC3 (%)	Ref
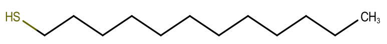 **1**	112-55-0	0.85	European Chemicals Agency (ECHA), Skin sensitisation study for CAS No. 112-55-0
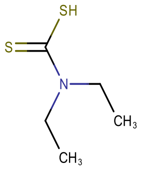 **2** (as Na salt)	148-18-5	1.66	Chipinda et al, Contact Dermatitis, 2008, 59, 79-89
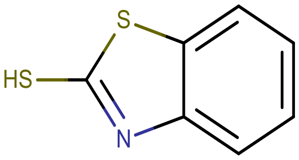 **3**	149-30-4	4.27	De Jong et al, Toxicological Sciences, 2002, 66, 226-232; Gerberick et al, Dermatitis, 2005, 16, 157-202; NTP Interagency Center for the Evaluation of Alternative Toxicological Methods (NICEATM), LLNA study for CAS No. 4
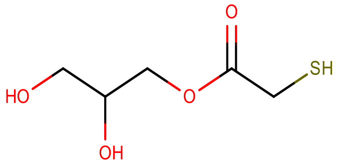 **4**	30618-84-9	4.66	NTP Interagency Center for the Evaluation of Alternative Toxicological Methods (NICEATM), LLNA study for CAS No. 30618-84-9, 2013
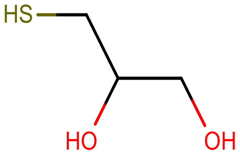 **5**	96-27-5	3.6	NTP Interagency Center for the Evaluation of Alternative Toxicological Methods (NICEATM), LLNA study for CAS No. 96-27-5, 2013

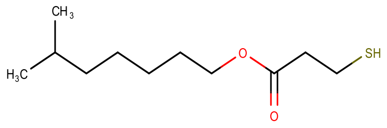<br>**6**	30374-01-7	8.2	European Chemicals Agency (ECHA), Skin sensitisation study for CAS No. 30374-01-7
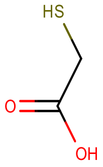<br>**7**	34316-71-7, 5421-46-5	5.33	Burnett et al, International Journal of Toxicology, 2009, 28, 68-133; SCCS (Scientific Committee on Consumer Safety), Opinion on CAS No. 5421-46-5, SCCS/1520/13
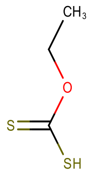<br>**8 (Na salt)**	30374-01-7	7.28	NTP Interagency Center for the Evaluation of Alternative Toxicological Methods (NICEATM), LLNA study for CAS No. 140-90-9, 2013
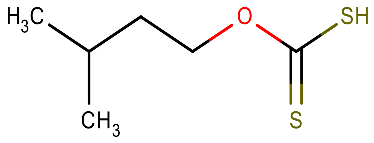<br>**9** (K salt tested)	928-70-1	10.8	European Chemicals Agency (ECHA), Skin sensitisation study for CAS No. 928-70-1
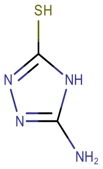<br>**10**	16691-43-3	8.37	NTP Interagency Center for the Evaluation of Alternative Toxicological Methods (NICEATM), LLNA study for CAS No. 16691-43-3, 2013
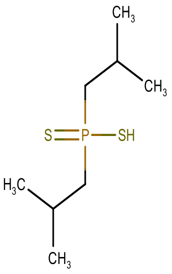	13360-78-6	18.1	European Chemicals Agency (ECHA), Skin sensitisation study for CAS No. 13360-78-6

**11** (sodium salt)			
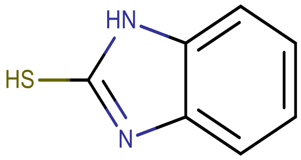<br>**12**	583-39-1	14.7	De Jong et al, Toxicological Sciences, 2002, 66, 226-232
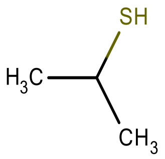<br>**13**	75-33-2	75.5	European Chemicals Agency (ECHA), Skin sensitisation study for CAS No. 75-33-2

Table S3. Phosphorus nucleophiles

Structure	CAS no.	EC3 (%)	Ref
$(PhO)_3P$<br>**14**	101-02-0	1.4	European Chemicals Agency (E18CHA), Skin sensitisation study for CAS No. 101-02-0
$(Me_2CH(CH_2)_7O)_3P$<br>**15**	25448-25-3	20	European Chemicals Agency (ECHA), Skin sensitisation study for CAS No. 16
$(Me_2CH(CH_2)_7O)_2POPh$<br>**16**	25550-98-5	41	Drewe et al. *Contact Dermatitis* **2017**, *76* (5). https://doi.org/10.1111/cod.12704.
$(Me_2CH(CH_2)_{10}O)_3P$<br>**17**	77745-66-5	92.1	European Chemicals Agency (ECHA), Skin sensitisation study for CAS No. 77745-66-5
$(EtO)_3P$<br>**18**	122-52-1	19/20 in GPMT (5% injection induction, 100% topical induction, 100% challenge). https://echa.europa.eu/da/registration-dossier/-/registered-dossier/6662/7/5/2	

Table S4. Carbon centered nucleophiles

Compound number	Structure	CAS no.	EC3 (%)	Ref
**20**	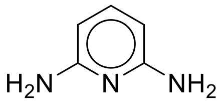	141-86-6	0.25	*a*
**19**	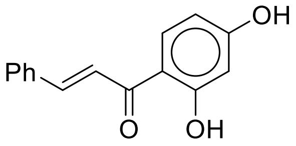	1776-30-3	0.49	*b*
**22**	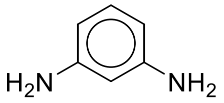	108-45-2	0.49	*c*
**23**	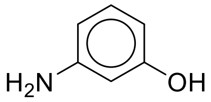	591-27-5	1.72	*d*
**24**	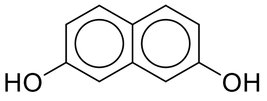	582-17-2	2.8	*e*
**25**	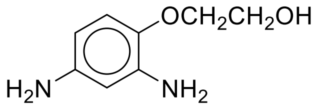	66422-95-5	3.2	*f*
**21**	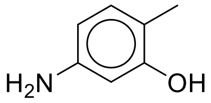	2835-95-2	3.4	*g*
**27**	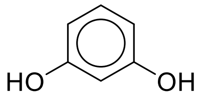	108-46-3	3.45	*h*
**26**	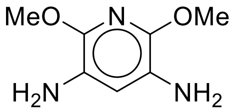	56216-28-5	4.07	*i*
**25**	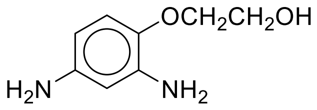	66422-95-5	4.35	*j*
**28**	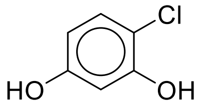	95-88-5	5.8	*k*
**29**	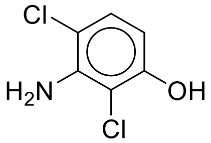	608-25-3	16.8	*l*
**30**	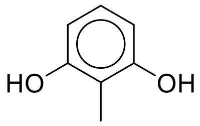	608-25-3	50	*m*

References to table S4

a SCCS (Scientific Committee on Consumer Safety), Opinion on CAS No. 141-86-6, SCCS/1503/13

b NTP Interagency Center for the Evaluation of Alternative Toxicological Methods (NICEATM), LLNA study for CAS No. 1776-30-3, 2013

c Gerberick et al, Dermatitis, 2005, 16, 157-202

d European Chemicals Agency (ECHA), Skin sensitisation study for CAS No. 591-27-5; Gerberick et al, Dermatitis, 2005, 16, 157-202

e SCCS (Scientific Committee on Consumer Safety), Opinion on CAS No. 582-17-2, SCCS/1366/10

f SCCS (Scientific Committee on Consumer Safety), Opinion on CAS No. 66422-95-5, SCCS/1367/10

g NTP Interagency Center for the Evaluation of Alternative Toxicological Methods (NICEATM), LLNA study for CAS No. 2835-95-2, 2013; SCCP (Scientific Committee on Consumer Products), Opinion on CAS No. 2835-95-2, SCCP/1001/06

h Basketter et al, Contact Dermatitis, 2007, 56, 196-200; Basketter et al, Food and Chemical Toxicology, 1994, 32, 543-547; Basketter et al, Food and Chemical Toxicology, 1998, 36, 327-333; European Chemicals Agency (ECHA), Skin sensitisation study for CAS No. 108-46-3; Gerberick et al, Dermatitis, 2005, 16, 157-202; Gerberick et al, Toxicological Sciences, 2007, 97, 417-427; NTP Interagency Center for the Evaluation of Alternative Toxicological Methods (NICEATM), LLNA study for CAS No. 108-46-3, 2013; Roberts et al, Chemical Research in Toxicology, 2007, 20, 1019-1030; SCCS (Scientific Committee on Consumer Safety), Opinion on CAS No. 108-46-3, SCCS/1270/09; Schneider and Akkan, Regulatory Toxicology and Pharmacology, 2004, 39, 245-*255*

i SCCP (Scientific Committee on Consumer Products), Opinion on CAS No. 56216-28-5, SCCP/0908/05

j Kern et al, Dermatitis, 2010, 21, 8-32; SCCS (Scientific Committee on Consumer Safety), Opinion on CAS No. 66422-95-5, SCCS/1367/10

k SCCS (Scientific Committee on Consumer Safety), Opinion on CAS No. 95-88-5, SCCS/1224/09

l SCCP (Scientific Committee on Consumer Products), Opinion on CAS No. 61693-43-4, SCCP/1205/08

m European Chemicals Agency (ECHA), Skin sensitisation study for CAS No. 608-25-3

Supporting information 3. Manual calculation of logP for cardanols, cardols and anacardic acids

In the manual method each CH_2 group contributes 0.54 to logP (sum of 2H at 0.23 each and 1C at 0.2, with a bond factor of -0.12) whereas in the computerised version 0.195 is used for C and 0.227 for H, giving a CH_2 group contribution of 0.529. There is also a difference in how double bonds are treated. In the manual version logP is calculated for the saturated analogue and then -0.55 is applied for each double bond, whereas in the computerised version all the fragment values are added (so 2H less for each double bond) and -0.03 is applied for the double bond. So in effect ClogP for a monounsaturated compound is less than that of the saturated analogue by 0.484. as compared with 0.55 in the manual method*.

Starting from measured logP of toluene listed in Hansch and Leo 1979
Toluene, logP =2.69
Insert 14 CH_2 at 0.54 each to give 10.25 as the calculated logP for n-pentadecylbenzene
For Cardanol (saturated) remove 1 H (subtract 0.23) and add 1 OH (add -0.44):
LogP for cardanol (sat) = 10.25-0.23-0.44 = 9.58
This logP value for cardanol (sat.) is subtracted from 11 to give the logPadj value of 1.42
For cardol (sat.), remove 1 H from cardanol (sat.) and add 1 OH:
logP for cardol (sat.) = 9.58-0.23-0.44 = 8.91; logPadj = 11-8.91 = 2.09
For anacardic acid (sat.), from cardanol (sat.) remove 1 H (0.23), add 1 CO_2^- (-4.13) and add 1.00 for an intramolecular H-bond to give logP = 6.22. Subtract from 11 for logPadj = 4.78
For each double bond, subtract 0.55 from the saturated analogue
Anacardic acid monounsat. logP = 5.67; logP adj = 5.33
Anacardic acid, di-unsat, logP = 5.12
Anacardic acid, tri-unsat, logP = 4.57
*Neither the manual nor the computerised method for logP makes any distinction between Z and E configurations. We do not know whether this reflects reality or a lack of relevant experimental data. For long chain unsaturated compounds we might expect Z isomers to be less hydrophobic as a result of water-sharing between the two chains that are *cis* to each other (analogous to the effect modelled by position-dependent branching factors in surfactants like alkylbenzenesulfonates). In the mono- and di- unsaturated compounds considered here the double bonds are Z (in the tri-unsaturated the third double bond is terminal). If the Z configuration does reduce the hydrophobicity relative to the E isomer, the effect would be to make the unsaturated cardanols and cardols more potent than our calculations suggest, and to

make the unsaturated anacardic acids less potent. The magnitude of these effects are unlikely to be sufficient to alter the QMM predictions that anacardic acid and cardol should be strong sensitizers and cardanol much weaker.
